# The intervention effect of mental health knowledge integrated into ideological and political teaching on college students’ employment and entrepreneurship mentality

**DOI:** 10.3389/fpsyg.2022.1002468

**Published:** 2022-10-04

**Authors:** Jiaming Zhu, Lanchuan Lei, Pengjv Wu, Bo Cheng, Xiu-lan Yang, Jing Fu, Zhaoxia Wu, Fangcheng He

**Affiliations:** ^1^School of Foreign Languages and Literatures, Chongqing University of Education, Chongqing, China; ^2^School of Literature and Communication, Chongqing Three Gorges University, Chongqing, China; ^3^School of Basic Medical Sciences, Yangtze University, Jingzhou, Hubei, China; ^4^School of Mathematics Physics and Big Data, Chongqing University of Science and Technology, Chongqing, China

**Keywords:** college students from divorced families, healthy personality, ideological education, mental health education, combination approach, employment mentality

## Abstract

In order to analyze the intervention effect of integrating mental health knowledge into ideological and political teaching on college students’ employment and entrepreneurship mentality, this paper proposes a study to predict the effect of integrated intervention. This research mainly investigates the ideological and psychological conditions of college students from divorced families through questionnaires, compares and analyzes the classification and statistical results of the survey data between groups and within groups, and analyzes the reasons for the ideological and psychological problems of college students. The experimental results show that 30% of college students from divorced families and college students from non-divorced families responded that they do not feel comfortable in places with many people, and the difference between the groups is not significant. Regarding the concept of entrepreneurship, 64.63% of college students from divorced families in urban areas believe that entrepreneurship is a form of learning and should be encouraged. 63.27% of college students from divorced families in rural areas believe that learning should be the first priority and that a business should not be started. 20.41% of college students from divorced families in rural areas and 25.61% of college students from divorced families in urban areas believe that because entrepreneurship provides economic income, it can reduce the burden on families, but the difference is not obvious. In short, this study can provide reference for the ideological and psychological status of college students from divorced families.

## Introduction

In China’s current society, the problem of college graduates having difficulty finding employment has become the focus of social attention. The employment of college graduates is not only related to students’ personal futures, but is also related to the happiness and stability of their families, as well as the stability of the country and social harmony. So, what are the problems that make it difficult for Chinese college graduates to obtain employment? Many people say that due to the expansion of college enrollment, the number of college students is higher than the social demand for talent, that is, supply exceeds demand (Kranz et al., 2021). Since 1999, China has continuously expanded the enrollment of colleges and universities, and the difficulty of College Students’ employment reflects many problems. It is not simply considered a social problem, but is more of a comprehensive reflection of the orientation of talent training goals in Colleges and universities and the low competitiveness of college students themselves. The development of higher education is no longer the previous ivory tower “elite education” model, but has gradually transitioned to a new stage of “popular education” (Bashir et al., 2021). The social evaluation orientation of college students, the talent training mode of colleges and universities, and the orientation of College Students’ values have not been transferred from “elite education” to the educational concept of “popular education” in this time. This is one of the root causes of the current difficulties in the employment of college students. The expansion of college enrollment has brought new problems and opportunities to college students, as well as new challenges to moral educators in Chinese colleges and universities.

**FIGURE 1:**
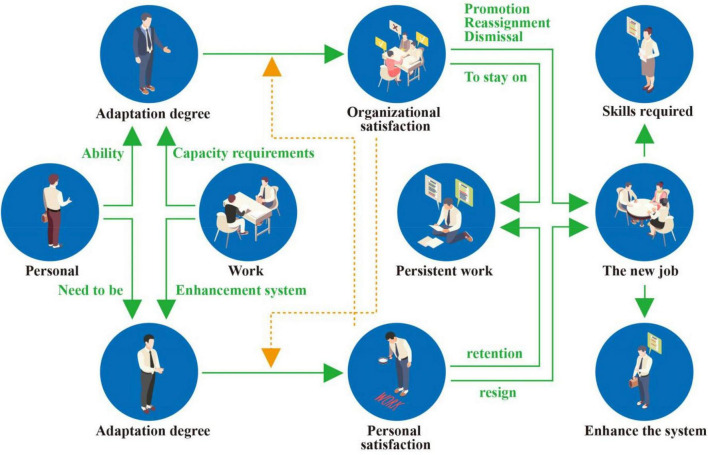
Employment process.

With the increasingly severe employment situation, the competition and challenges faced by college graduates will increase correspondingly, and their cruelty and intensity will also intensify, which will inevitably bring great pressure to their hearts and spirits. In recent years, it has been common for college graduates to commit suicide because they cannot find a job, which is cause for alarm. it is necessary to provide quality psychological counseling and humanistic care in the employment of college students to avoid negative consequences (Deo et al., 2020). Therefore, the question of how to bring attention to the role of mental health education in college students’ career planning and help college students achieve smooth employment and healthy development is a new research topic we are facing. As shown in Figure 1, good employment mentality and good entrepreneurial mentality are the guarantees of successful employment. Therefore, college students’ employment and entrepreneurship mental health education has attracted attention from many perspectives. The integration of mental health knowledge into ideological and political teaching can give full play to the supporting role of mental health education in college students’ career planning. At the same time, it can also promote the realization of higher education goals and the implementation of the “people-oriented” concept.

## Literature review

Rui et al. (2020) pointed out that mental health education includes the cultivation of good psychological quality and the prevention and treatment of mental diseases, which complement each other. The educational content of optimizing students’ psychological quality includes: intelligent development education, cultivation of non intelligent factors, harmonious interpersonal relationship education, environmental adaptation education, and healthy personality education. The education content of preventing mental disease includes: mental health knowledge education, frustration education, and mental disease prevention education. The research of Juurlink et al. (2020) shows that the content of mental health education includes propagating the concept of modern health, explaining the methods of achieving mental health, including self acceptance, developing social interaction, and learning psychological adjustment, and introducing psychological counseling knowledge. Sanwald et al. (2022) pointed out that college students’ mental health includes self-awareness and self-improvement, emotion and emotional regulation, frustration, and stress. Personality optimization will exercise college students’ learning psychology, college students’ interpersonal psychology, college students’ love psychology, network psychology and adjustment, college students’ career planning, and college students’ common psychological obstacles and prevention. Wang (2020) proposed that ideological and political education in colleges and universities should always adhere to the guidance of Xi Jinping’s Thought on Socialism with Chinese Characteristics for a New Era, adhere to the combination of theoretical innovation and practical innovation, and improve the timeliness of ideological and political education for college students. In order to better non-divorced the fundamental task of colleges and universities, the foundation of the establishment of schools should be to establish morality and cultivate people (Wang, 2020). Kummer et al. (2020) proposed that ideological and political work should run through the whole process of education and teaching, and recognized the model of teaching people throughout the whole process and teaching the whole science, which is a perfect and systematic model of popular science teaching. This is the innovation of General Secretary Xi Jinping in the cultivation of socialist workers and graduates in colleges and universities (Kummer et al., 2020). Davoudian et al. (2021) proposed that the understanding and handling of the value relationship between the “small self” and “big self” is the fundamental problem of value guidance. Zhou et al. (2022) said that the school’s philosophy and politics course is an important course to help students form a world outlook and an outlook on life and values, arm students with Marxist theory, and support social builders and achievers. Conley et al. (2022) proposed that a distinctive feature of ideological and political education in Chinese colleges and universities in the development process is to adhere to the guidance of morality and talent, and focus on constantly adhering to and improving the party’s leadership, occupying the ideological front, and strengthening ideological and theoretical education and value guidance. Tement et al. (2020) proposed that school should “scientifically analyze the development process and characteristic level of ideological and political education since the founding of New China, summarize historical and historical capabilities, and provide historical materials for ideological and political education in the new era.”

This study mainly investigates the ideological and psychological status of college students from divorced families from the aspects of ideal and belief, employment tendency, marriage and love outlook, independence, social psychology, emotional state, and psychological appeal. The classification results of the survey data are compared and analyzed between groups (college students from divorced families and college students from non-divorced families) and within groups (mainly involving the differences between male and female students, urban and rural differences, grade differences, etc. among college students from divorced families). The causes of ideological and psychological problems of college students from divorced families are analyzed through detailed data comparison and combining the relevant knowledge of pedagogy, psychology, and sociology.

## Materials and methods

### Scheme implementation methods and means

For the implementation of the survey plan on the ideological and psychological status of college students from divorced families, the following methods are mainly used:

Comparative analysis, is to understand the content and laws of things by comparing targets and making accurate evaluations. Comparative analysis is often used to compare two related data analyses, showing and explaining the size, level, speed, and integration of multiple relationships of the research object. In this study, the survey groups were compared between groups and within groups, and the survey results were compared and analyzed: (1) inter group survey: the data from the statistical results of college students from divorced families and college students from non-divorced families were compared in terms of Ideological and psychological status and (2) intra group investigation: compare the ideological and psychological status of college students from divorced families with different genders, grades, and growth backgrounds (urban/rural) (Mace et al., 2021). Interview refers to psychological research to understand the psychology and behavior of the interviewee through face-to-face conversation between the interviewer and the interviewee. This study selected several college students from divorced families from different schools to conduct face-to-face interviews, through which the most direct, in-depth, reliable, and effective data were obtained.

For specific cases, research and analysis can be carried out to excavate reasons, reveal laws, and explore ways and methods to solve problems.

The data results of this study can be analyzed from multiple disciplines and fields. On this basis, this paper analyzes the causes of the ideology of college students from divorced families through the integration of psychological barriers from college to a career (Sanders et al., 2020).

### Questionnaire and data statistical results

The practical research of this study is mainly carried out by means of questionnaire, which was designed according to the comprehensive analysis of relevant data. An anonymous questionnaire survey was planned for full-time part-time college students from divorced families and college students from successful families in five universities in a city (in order to make the results of the questionnaire more reliable, this questionnaire is designed to investigate psychological counselors’ thoughts and feelings). The questionnaire used to investigate the ideological and psychological status of college students was mainly designed from seven dimensions: (1) ideal and belief (1–3 questions); (2) employment tendency (4–9 questions); (3) love and marriage (10–11 questions); (4) independence (12–14 questions); (5) social psychology (15–23 questions); (6) emotional state (24–30 questions); and (7) psychological appeal (31–35 questions). The questionnaire used to investigate college counselors’ views on the ideological and psychological status of college students from divorced families mainly confirms the above seven dimensions and improves the reliability of the survey results.

This survey distributed and collected anonymous questionnaires to 800 college students from five full-time undergraduate colleges in a city. According to preliminary statistics, among the 800 questionnaires for college students’ ideology, 796 were recovered, 789 were valid, and the recovery rate was 99.12%. Among the 789 valid questionnaires, there were 153 college students from single-parent families, 131 college students from divorced families, and 636 college students from non-divorced families. In order to make the results of the comparative analysis more reliable, 131 questionnaires from 636 college students from graduate families were selected and compared with 131 statements made by college students from divorced families. Among the 50 questionnaires on Counselors’ views on the ideological and psychological status of college students from divorced families, 50 questionnaires were recovered and 50 were valid, with an effective recovery rate of 100%.

SPSS 21.0 statistical software was used to process the data, and t-test was used for comparison. The counting data was tested by χ^2^ test, with *P* < 0.05 as the difference, which was statistically significant (Wattick et al., 2021).

The mathematical definition formula of factor correlation analysis using chi square test is as follows (Eq. 1):


(1) $$\chi^{2}=\sum_{i=1}^{r}\sum_{j=1}^{c}\frac{{\left(f_{ij}^{0}\,-\,f_{ij}^{e}\right)}^{2}}{f_{i\,j}^{e}}$$

where, *r* is the number of rows in the contingency table; *c* is the number of columns in the contingency table; $f_{ij}^{0}$ is the observation frequency; $f_{ij}^{e}$ is the expected frequency.

The formula for calculating the expected frequency *f^e^* is as follows (Eq. 2):


(2) $$f^{e}=\frac{R\,T}{n}*\frac{C\,T}{n}*n=\frac{R\,T*C\,T}{n}$$

where, *RT* is the total of line observation frequencies; *CT* is the total of column observation frequencies.

From the chi-square statistic obtained from the above formula, it can be seen that if the frequency and measurement are to be the same, the chi-square statistic is the smallest and is 0. The larger the difference between the expected frequency and the observed frequency, the larger the chi-square statistic that can be obtained and the higher the correlation.

## Results and analysis

Using the seven dimensions of the questionnaire design, the data of 131 questionnaires on the thoughts and feelings of college students from divorced families and 131 questionnaires on the thoughts and feelings of college students from non-divorced families were compared between groups. The comparative analysis results are shown in Table 1.

**TABLE 1:** Some survey data on ideals and beliefs.

Percentage of people who choose this item in the survey of this group		College students from divorced families (%)	College students from non-divorced families (%)
Foundation of ideal establishment	Parents’ requirements, family pressure	64.89	39.69
	Interest	24.43	34.35
	Social/market demand	9.16	21.37
	No ideal	1.53	4.58
Who do you want to be in the future	Average person	45.04	39.69
	A rich man	28.24	19.08
	People who realize self-worth	24.43	37.40
	Indifferent	2.29	3.82

The statistical data are shown in Table 1. It can be seen from the data results that more than half of the college students from divorced families choose to build the ideal foundation from parental requirements and family pressure. In contrast, college students from non-divorced families focus on parental requirements, family pressure, interests, and social or market needs; As for what kind of people they hope to become in the future, a horizontal comparison between the two groups found that college students from divorced families choose that they hope to become rich more than those from non-divorced families, and college students from non-divorced families choose that they hope to become people who realize their self-worth more than those from divorced families (Agans et al., 2020; Hager et al., 2022).

The investigation of employment tendency can indirectly reflect the psychological pressure and ideological status of college students to a certain extent. Neither college students from divorced families nor college students from non-divorced families choose not to agree with taking part-time jobs (Negash et al., 2022; Tele et al., 2022). The survey results of other employment tendencies are shown in Table 2. According to the survey data, college students from divorced families pay more attention to wages for employment than college students from non-divorced families, while college students from non-divorced families pay more attention to talents and working environments when seeking employment than college students from divorced families; According to the survey of their views on entrepreneurship, college students from divorced families were more likely to believe that entrepreneurship is another kind of learning and that more people should be encouraged to start a business and have economic income to reduce the burden on their families than college students from non-divorced families. College students from non-divorced families choose to study first. Those who chose not to start a business were relatively higher in the group of students from non-divorced families than from divorced families, with more than half of students from non-divorced families choosing to study first. According to the survey on when to start employment preparation, college students from divorced families generally choose earlier than college students from non-divorced families.

**TABLE 2:** Partial survey data on employment tendency.

Percentage of people who choose this item in the survey of this group		College students from divorced families (%)	College students from non-divorced families (%)
What is the most important thing for future employment	Play of talents	22.90	34.35
	Wages	47.33	30.53
	Work environment	21.37	29.77
	Other	8.40	5.34
Views of college students on Entrepreneurship	Study first, don’t start a business	30.53	58.78
	It is another kind of learning, and entrepreneurship should be encouraged	45.80	31.30
	Having economic income can reduce the burden on the family	23.66	9.92
When does employment preparation begin	Freshman	20.61	7.63
	Sophomore	55.73	23.66
	Junior	22.90	55.73
	Senior	6.87	12.98

College Students’ views of love and marriage are easily affected by family education, environment, and other factors. College students’ views of love and marriage refer to college students’ basic views on premarital love, marriage life, and the process of love and marriage. The concept of marriage and love formed at the university level will directly affect the life of college students, and it is also related to the future happiness of college students. A bad view of marriage and love will inevitably bring some ideological, psychological, and behavioral deviations to college students. The survey data on how college students from divorced families and college students from non-divorced families view campus love are shown in Table 3. More than half of college students from non-divorced families choose a suitable partner, which is acceptable. The number of divorced families who choose this option only accounts for nearly 1/4, and is not significant. The number of college students from divorced families who choose it naturally is also significantly higher than that from non-divorced families. A small number of people in both groups choose not to develop relationships, but the number of college students from divorced families who selected this response is more than that from non-divorced families. As for how to treat the relationship between love and marriage, nearly 80% of college students from non-divorced families chose that response that love as the basis of marriage, and marriage is the continuation of love. Relatively speaking, nearly 60% of college students from divorced families choose this option. Compared with college students from divorced families, those who responded that they only want to fall in love and do not want to get married are higher than those from non-divorced families.

**TABLE 3:** Survey data on marriage and love.

Percentage of people who choose this item in the survey of this group		College students from divorced families (%)	College students from non-divorced families (%)
How to treat campus love	If there is a suitable object, it is acceptable	31.30	61.83
	Unrealistic, because I will break up sooner or later after graduation	6.11	2.29
	It doesn’t matter. Let it be	47.33	20.61
	Unwilling to develop a relationship	6.11	3.82
	Longing for love	8.40	11.45
How to treat love and marriage	Love not for marriage is all hooliganism	21.37	6.87
	Love is love, marriage is marriage	5.34	6.87
	I just want to fall in love, not get married	16.03	7.63
	Love is the foundation of marriage, and marriage is the continuation of love	57.25	78.63

In terms of venting when facing pressure and annoyance, more than 70% of college students from divorced families chose not to say it in their hearts or to find a suitable way to vent such ascrying or exercising, while college students from non-divorced families were more likely to choose to tell others. The survey results of the time required to adapt to college life and who to ask for help when encountering difficulties are shown in Table 4. According to the survey data, more than half of the college students from non-divorced families need 2–6 months to adapt to college life. The number of college students from divorced families who need more than 6 months is less than that of college students from divorced families, while the number of college students from divorced families who adapt to college life within one month is relatively more than that of college students from non-divorced families. This is noteworthy; When encountering difficulties, college students from divorced families responded that they focus on their own decision rather than consult with others or ask friends for help, while more than half of college students from non-divorced families responded that they ask friends for help.

**TABLE 4:** Partial survey data on independence.

Percentage of people who choose this item in the survey of this group		College students from divorced families (%)	College students from non-divorced families (%)
Time to adapt to college life	Within 1 month	31.30	19.08
	2–6 months	30.53	64.89
	6 months to 1 year	13.74	11.45
	More than 1 year	21.37	3.82
	Never adapted	3.05	0.76
Who can I ask for help in case of difficulties	Decide by yourself and don’t talk to others	38.17	15.27
	Parent	19.08	20.61
	Friend	30.53	54.20
	Teacher	1.53	3.82
	Surf the internet	8.40	5.34
	Other	2.29	0.76

Studies have shown that the major stress of family breakdown will lead to teenagers from divorced families being troubled by negative emotions for a long time, become insecure and closed, lack a sense of security and trust in people, and thus affect their social interaction (Lindow et al., 2020). These studies have investigated whether or not the students want to join in with conversations, want to participate in the sports meetings or theatrical performances, like talking with others, and want to take revenge immediately after being spoken ill of. The survey results of college students from divorced families and college students from non-divorced families are almost the same. Most of them show that they want to join the conversation, do not hate to participate in the sports meeting or theatrical performance, do not dislike talking with others, and do not want to take revenge for being spoken ill of. The results of other social psychology surveys are shown in Table 5. In terms of whether to serve as student cadres, half of college students from divorced families do not serve as student cadres, and about 1/4 of college students from non-divorced families do not serve as student cadres; In a survey assuming that family conditions are difficult, when choosing how to make an impression, college students from divorced families mainly choose excellent grades and perseverance, while college students from non-divorced families are relatively more likely to choose people with an open mind; In both groups, fewer people responded that they feel that their classmates speak ill of themselves behind their backs, but college students from divorced families chose this response a little more; The number of college students from divorced families who think they have few friends is also higher than that of college students from non-divorced families; Finally, about 30% of college students from divorced families and those from non-divorced families responded that they do not to adapt in the survey on whether they do not adapt in places with a large number of people, and there is not a great difference.

**TABLE 5:** Partial survey data of social psychology.

Percentage of people who choose this item in the survey of this group		College students from divorced families (%)	College students from non-divorced families (%)
Whether to be a student cadre	Work in class	18.32	29.01
	Work in the society	28.24	38.17
	No position	53.44	32.82
How to make an impression	Smart brain	9.92	23.66
	Excellent results	39.69	27.48
	An indomitable character	35.11	12.98
	An open-minded mind	15.27	35.88
I feel that my classmates speak ill of me behind my back	Yes	9.92	1.53
	No	90.08	98.47
I have few friends	Yes	24.43	7.63
	No	75.57	92.37
I am not suited for places with many people	Yes	31.30	29.01
	No	68.70	70.99

When investigating how students react to situations in which blame can be placed, more than 90% of the results of college students from divorced families and college students from non-divorced families tend not to think they are at fault. See Table 6 for statistics of differences in emotional state survey results. In terms of emotional survey, about 75% of college students from divorced families responded that they felt changeable and depressed, while half of college students from non-divorced families responded that they felt emotionally stable, and some reported being changeable. In terms of personality, college students from divorced families are significantly more likely than those from divorced families to report that they are outgoing, cheerful, and find it easy to communicate, while college students from divorced families are significantly more likely than those from non-divorced families to report that they are calm, love to be alone, and talk less. Another question asked students whether they still feel lonely even surrounded by people, always think they are bad when being criticized, and whether they want to go to a distant place alone. College students from divorced families chose yes more than those from non-divorced families; As for whether they feel inferior due to family changes, 21.37% of divorced families choose yes, while only 1.53% of college students from non-divorced families choose yes.

**TABLE 6:** Partial survey data of emotional state.

Percentage of people who choose this item in the survey of this group		College students from divorced families (%)	College students from non-divorced families (%)
Your emotions are often as follows	High spirits	0.00	5.34
	Be down in spirits	21.37	7.63
	Changeable	54.96	35.11
	Relatively stable	23.66	51.91
Do you feel your character is:	Outgoing and easy to communicate	16.03	46.56
	Keep calm	35.88	21.37
	Love to be alone and talk less	36.64	12.98
	Other	11.45	19.08
Do you still feel lonely when surrounded by people	Yes	21.37	3.82
	No	78.63	96.18
I always think I’m bad when I’m criticized	Yes	12.98	1.53
	No	87.02	98.47
Do you want to go to a distant place alone	Yes	16.79	3.82
	No	83.21	96.18
Will you feel inferior because of family changes	Yes	21.37	1.53
	No	78.63	98.47

The understanding of psychological demands can reflect the psychological needs of college students from divorced families in some aspects. As for the most distressing problems in college, the survey results of college students from divorced families are mainly manifested in high school costs, financial difficulties, employment pressure, and other options, while the survey results of college students from non-divorced families are mainly manifested in employment pressure, lack of interest in their majors, and love problems. Other findings on psychological appeal are shown in Table 7. There is little difference between the survey responses of the two groups regarding whether they want help from others. Regarding whether it is difficult to deal with difficult things, more college students from non-divorced families responded “yes” than from divorced families. In terms of the public opinion of divorced families, more than half of college students from divorced families think that some aspects are biased but not obvious, some people think that there is no discrimination and no special concern, more people are not clear about the public opinion of non-divorced families, and some people think that some aspects are biased, but not obvious; Finally, about half of divorced families think they have some concern about social policies and systems, while about 1/6 of non-divorced families think they have some concern, and nearly 70% of people are not very clear.

**TABLE 7:** Partial survey data of psychological appeal.

Percentage of people who choose this item in the survey of this group		College students from divorced families (%)	College students from non-divorced families (%)
Do you want help from others	Yes	58.02	61.83
	No	41.98	38.17
Is it difficult to deal with difficult things	Yes	32.82	58.02
	No	67.18	41.98
Public opinion on divorced families	Very concerned	3.82	0.76
	No discrimination or special concern	22.14	8.40
	Biased in some aspects, but not obvious	54.96	35.88
	There is obvious discrimination	5.34	12.98
	Not too clear	13.74	41.98
Social policies and systems on divorced families	Very concerned	2.29	0.76
	There are some concerns	54.96	21.37
	No special care	16.03	9.92
	Not too clear	26.72	67.94

**FIGURE 2:**
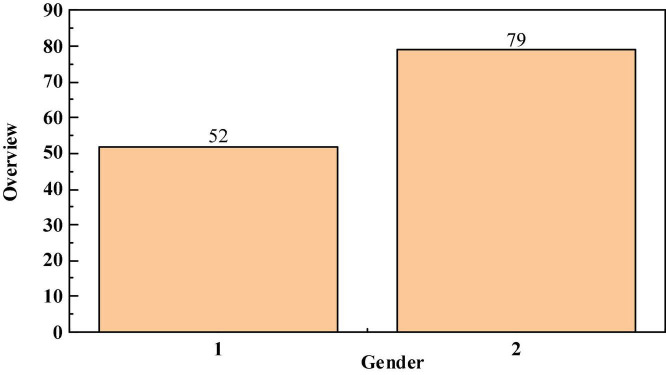
Overview of gender classification of college students from divorced families.

**FIGURE 3:**
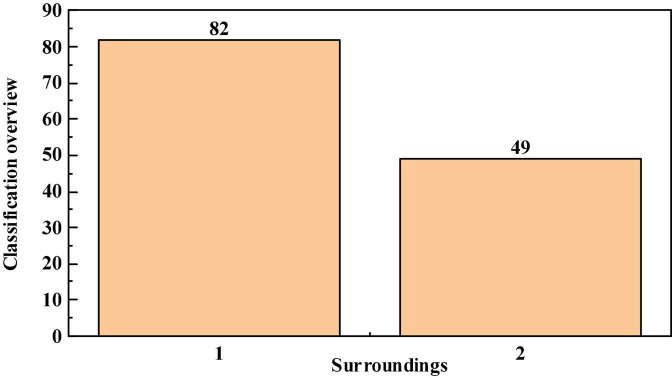
Overview of the growth environment classification of college students from divorced families.

The questionnaires collected from 131 college students from divorced families were compared and analyzed within the group from two aspects: gender and growth environment. According to statistics, the profile of college students from divorced families surveyed is shown in Figures 2, 3.

According to the division of gender and growth environment, college students from divorced families are compared and analyzed within the group from the seven dimensions of the questionnaire design, because not every classification of the comparative analysis results can see obvious differences. Therefore, only some statistical results that are meaningful for research and analysis are described and analyzed. The statistical results of comparative analysis are as follows.

According to the survey on the foundation of ideal and belief building of college students from divorced families, more than half of college students from divorced families responded that their foundation for beliefs and ideals comes from the requirements of their parents and family pressure, and 24.43% responded that they come from their own interests. For these two options, the differences within the group are mainly reflected in gender and growth environment. The specific statistical data are shown in Table 8. It can be seen from the Table that more than 80% of male college students from divorced families responded that the ideals are founded from parental requirements and family pressure. Relatively speaking, although half of girls choose this option, 25/79 girls chose that their ideals were founded from their interests than boys; However, college students from divorced families in rural areas responded that they establish their ideals on the basis of parents’ requirements and family pressure, which is obviously much more than college students from divorced families in cities. Accordingly, the number of respondents who chose that the foundation of ideals comes from their own interests was much less in rural areas than cities (Xu et al., 2021).

**TABLE 8:** Intra group comparative data on ideals and beliefs of college students from divorced families.

The number of people of each type who choose this option (unit: person)	Gender		Growth environment	
	Boys (52)	Girls (79)	City (82)	Country (49)
The foundation of ideal comes from parents’ requirements and family pressure	43	42	45	40
The foundation of ideal comes from interest	7	25	28	4

The relevant survey options were selected in terms of employment tendency. It can be seen that there were differences within the group, and the comparative data are shown in Table 9. It can be seen from the table that in terms of employment tendency, gender differences are mainly reflected in the fact that female college students from divorced families tend to think that learning should be prioritized and over business, compared with male college students. Compared with female college students, male college students from divorced families tend to think that entrepreneurship is a kind of learning and worth encouraging, and also believe that entrepreneurship with economic income can reduce the burden on their families. The difference in growth environment is reflected in the fact that for college students from divorced families living in cities, more people value the display of talents and the working environment when they are employed. However, college students from divorced families living in rural areas are relatively less interested in these two aspects, and more in wages. As for the view of entrepreneurship, 64.63% of college students from divorced families living in cities think entrepreneurship is a kind of learning and worth encouraging, 63.27% of college students from divorced families living in rural areas think that learning is the priority and that a business should not be started, while 20.41% of college students from divorced families in rural areas and 25.61% of college students from divorced families in cities think that entrepreneurship has benefits in the form of economic income and can reduce the burden on their families, and the difference is not very obvious.

**TABLE 9:** Intra group comparative data on employment tendency of college students from divorced families.

The number of people of each type who choose this option (unit: person)	Gender		Growth environment	
	Boys (52)	Girls (79)	City (82)	Country (49)
Employment focuses on the exertion of talents	9	21	25	5
Pay attention to employment	30	32	30	32
Pay attention to the working environment in employment	8	20	22	6
Think that learning is first, and you shouldn’t start a business	9	31	9	31
I think entrepreneurship is a kind of learning and worth encouraging	19	41	53	7
Entrepreneurship has economic income, which can reduce the burden on the family	24	7	21	10

Differences in emotional love in the groups studied are often related to gender. In terms of falling in love while attending school, male college students in divorced families are more likely than female college students. From Table 10, it can be seen that about 30% of women from divorced families with higher education think that love without marriage is immoral, as opposed to about 10% of men from divorced families with higher education. Although the total number of college students from divorced families who only want to fall in love but do not want to get married is not large (21 out of 131 college students from divorced families chose this option), 18 of these 21 are female college students from divorced families, which deserves attention; There are still 57.25% of college students from divorced families who are more rational. They believe that love is the basis of marriage and marriage is the continuation of love. There is little difference within the survey group (Ajay et al., 2022b).

**TABLE 10:** Intra group comparison data of college students from divorced families in love outlook.

The number of people of each type who choose this option (unit: person)	Gender		Growth environment	
	Boys (52)	Girls (79)	City (82)	Country (49)
For campus love, it doesn’t matter, let it be	47	15	41	21
Think that love without marriage is all hooliganism	5	23	16	12
Love and marriage, just want to love, don’t want to get married	3	18	17	4
Love is the foundation of marriage, and marriage is the continuation of love	34	41	49	26

The statistical results of the investigation on the independence of college students from divorced families are shown in Table 11. In terms of the time to adapt to college life, it can be clearly seen that male college students from divorced families who adapt quickly in a short time (within 1 month) account for the majority, while female college students from divorced families who spend a long time and find it difficult to adapt to college life, and college students from divorced families living in rural areas, account for the majority; There is a slight difference between male and female college students from divorced families (36.54 and 39.24%, respectively) in the number of students who choose to solve problems independently, and there is also a slight difference between college students from divorced families in urban and rural areas (32.93 and 26.53%, respectively). Female college students from divorced families choose to seek help from their parents more than male college students from divorced families. Male college students from divorced families are more inclined to seek help from friends (Heydarpoor et al., 2020; Ashourizadeh et al., 2022). Among the 25 college students from divorced families who choose to seek help from their parents when they encounter difficulties, all of them are college students from divorced families born in cities and none of them live in rural areas.

**TABLE 11:** Intra group comparative data on the independence of college students from divorced families.

The number of people of each type who choose this option (unit: person)	Gender		Growth environment	
	Boys (52)	Girls (79)	City (82)	Country (49)
Adapt to college life within 1 month	29	12	30	11
2-6 months to adapt to college life	17	23	30	10
More than 1 year to adapt to college life	6	22	8	20
Solve difficulties by yourself	19	31	27	13
Ask parents for help in case of difficulties	8	17	25	0
Ask friends for help when you encounter difficulties	23	17	28	16

The intra group survey statistics of divorced college students on social psychology are shown in Table 12. First of all, nearly 60% of male college students from divorced families have participated in student cadres, while relatively few female college students have, at just under 40%. There is a greater difference in the number of student cadres between college students from divorced families living in cities and college students from divorced families living in rural areas. The number of college students from divorced families living in cities who have joined student cadres accounts for 60.98% of the total number of such groups. The corresponding data of college students from divorced families living in rural areas is 22.45%; Nearly half of the college students from divorced families living in cities prefer to make an impression with excellent grades, while only 24.49% of the college students from divorced families living in rural areas prefer to make an impression with indomitable character (accounting for 61.22% of this group), while only 19.51% of the college students from divorced families living in cities choose this option; In general, 32 college students from divorced families feel that they have few friends, of which 23 are girls and 24 live in cities, relatively more than boys and those who live in rural areas; Compared with college students from divorced families and college students from divorced families living in rural areas, more female college students from divorced families and college students living in cities feel that they will not adapt to places with many people (Zhao et al., 2019).

**TABLE 12:** Intra group comparative data on social psychology of college students from divorced families.

The number of people of each type who choose this option (unit: person)	Gender		Growth environment	
	Boys (52)	Girls (79)	City (82)	Country (49)
Number of student cadres	30	31	50	11
Tend to impress with excellent results	17	35	40	12
Tend to impress people with tenacious character	20	26	16	30
Tends to impress people with an open-minded mind	6	14	13	7
Think there are few friends	9	23	24	8
Don’t feel comfortable in a crowded place	25	16	19	22

The survey data on the differences among college students from divorced families in terms of emotion are shown in Table 13. In general, college students from divorced families choose far more people with changeable emotions than those who are depressed and stable. Among the 72 people who responded that they are often moody, 54 are girls, while nearly half of male college students from divorced families responded that they are emotionally stable, as opposed to only 12.66% of female students. In terms of personality, more than half of male college students from divorced families choose to keep their personality quiet, as opposed to only 25.32% of female college students. If we compare and analyze the growth environment, college students from divorced families living in rural areas choose to keep their personality quiet more than female college students. Among the 28 people who still feel lonely when they are surrounded by people, 21 are girls and 7 are boys; There are 28 college students from divorced families who feel inferior because of family changes. College students from divorced families living in cities have more choices than those living in rural areas (28.05 and 10.20% of this group, respectively).

**TABLE 13:** Intra group comparative data on emotional state of college students from divorced families.

The number of people of each type who choose this option (unit: person)	Gender		Growth environment	
	Boys (52)	Girls (79)	City (82)	Country (49)
Often depressed	13	15	18	10
Moody	18	54	43	29
Emotional stability	21	10	21	10
Personality is calm	27	20	22	25
Like to be alone and talk less with others	18	30	29	19
Still feel lonely when surrounded by people	7	21	15	13
Feel inferior due to family changes	11	17	23	5

The statistical results of the group survey on the psychological appeals of college students from divorced families are shown in Table 14. The number of female college students from divorced families who want help from others is significantly higher than that of male college students from divorced families, accounting for about 70% of this group (38.46% of male college students from divorced families) (Saeedikiya et al., 2021b; Zhan et al., 2021; Ajay et al., 2022a). From the perspective of growth environment, although 43 college students from divorced families living in cities (only 33 in rural areas) hope to get help from others, due to the fact that there are more people from cities in the survey group, the proportion of college students from divorced families living in cities is smaller, 52.44%, as opposed to 67.35% of college students from divorced families living in rural areas (Ebadi and Shiri Shahraki, 2010; Saeedikiya and Aeeni, 2020; Saeedikiya et al., 2021a). The number and proportion of female college students from divorced families are higher than that of male college students from divorced families. From the perspective of growth environment, the survey results are similar in terms of those who hope to get help from others. Although the number of college students from divorced families living in rural areas is small, the proportion is higher. Finally, 72 of 131 college students from divorced families think that social public opinion is biased against some aspects of divorced families, but it is not obvious, as well as 46.15% of boys and 60.76% of girls, and 62.20% of college students from divorced families living in cities and 42.86% of college students from divorced families living in rural areas.

**TABLE 14:** Intra group comparative data on psychological demands of college students from divorced families.

The number of people of each type who choose this option (unit: person)	Gender		Growth environment	
	Boys (52)	Girls (79)	City (82)	Country (49)
Hope to get help from others	20	56	43	33
Difficult things will not feel difficult to deal with	35	53	49	39
Believe that public opinion is biased against some aspects of divorced families, but it is not obvious	24	48	51	21

The most prominent psychological problems that parental divorce bring to college students from divorced families are personality and emotional problems, as well as the impact of ideological understanding. Emotion is an integral part of personality, and personality and thought are gradually formed on the basis of inborn environment. Therefore, even if college students from divorced families are affected by theirfamily environment and social environment in the process of growing up and have ideological and psychological bias, it is not inevitable. On the contrary, appropriate methods (such as the combination of ideological education and mental health education discussed in this study) can help them correct their personality, achieve better social adaptation and shape a healthy personality. Teachers of ideological and theoretical courses should update relevant educational concepts and educational content in a timely manner for college students from divorced families according to the aforementioned educational goals, educational content, and the combination of “two educations,” to improve mental health education awareness and fully consider divorced families in the teaching process. This will allow educators to meet the educational needs of college students, focus on the role of positive personality characteristics of college students from divorced families, integrate the above-mentioned educational concepts such as active attention and encouragement education, and cleverly adopt the relevant educational practice approach based on the combination of “two educations” based on the cultivation of healthy personalities in college students from divorced families.

The full-time teachers of mental health education already have a solid theoretical foundation in psychology and a solid level of business practice. In the process of cultivating healthy personalities for college students from divorced families, they should also strengthen basic ideological and theoretical education knowledge, and cooperate with other departments, institutions, and teachers to undertake psychological counseling related work, such as giving them psychological-related educational opinions when they hold relevant forums with counselors, class teachers, or teachers of ideological theory courses, and help them better use psychological knowledge in the classroom or outside of the classroom. In the communication of individuals, it can effectively alleviate and solve the ideological and psychological problems of college students from divorced families, improve the psychological quality of college students from divorced families, and strive to tap into their positive personality characteristics.

## Conclusion

The combination of ideological education and mental health education conforms to the typical personality characteristics of college students from divorced families, meets the internal needs of college students from divorced families’ ideological and psychological status quo, and becomes a good educational way to cultivate the healthy personalities of college students from divorced families. This study investigates the ideological and psychological status of college students from divorced families in terms of ideals and beliefs, employment tendency, marriage and love outlook, independence, social psychology, emotional state, and psychological demands. According to the research results, combined with the relevant literature of divorced college students, the positive and negative personality characteristics of this group can be summarized, which can be used to represent the ideological and psychological status of college students from divorced families, and also provide a reference for colleges and universities to formulate the personality training strategies of divorced college students. According to the analysis of the survey results, we try our best to meet the relevant personality training needs of college students from divorced families, and put forward the relevant educational ideas, educational practice, and institutional guarantee of organically combining ideological education and mental health education to cultivate the healthy personality of college students from divorced families, which provides reference and practical educational significance for the cultivation of healthy personality of college students from divorced families. Colleges and universities should strengthen the ideological and political education of graduates to ensure that the training of talents in colleges and universities better meets the actual needs of the society. Of course, the ideological and political education of graduates is by no means only a matter for the employment guidance department, nor is it only education for graduates, but an effective system that runs through the entire university education and involves the participation of all staff. Therefore, in order to deepen the research on the ideological and political education of graduates, it is necessary for everyone to explore and work together.

## Data availability statement

The original contributions presented in the study are included in the article/supplementary material, further inquiries can be directed to the corresponding author.

## Author contributions

JZ: data curation, writing—original draft, and writing—review and editing. LL: methodology. PW: project administration. BC: software and formal analysis. X-LY and JF: investigation, resources, and conceptualization. ZW and FH: supervision, validation, and visualization. All authors contributed to the article and approved the submitted version.
